# The nonlinear association between Body Roundness Index and left ventricular diastolic dysfunction in type 2 diabetes

**DOI:** 10.3389/fendo.2026.1709785

**Published:** 2026-02-25

**Authors:** Yanan Li, Zhiguo Wang, Yifei Ma, Nailong Yang

**Affiliations:** 1 Department of Endocrinology and Metabolism, The Affiliated Hospital of Qingdao University, Qingdao, China; 2 Department of Medicine, Qingdao University, Qingdao, China

**Keywords:** Body Roundness Index, cross-sectional study, left ventricular diastolic dysfunction, non-linear association, type 2 diabetes mellitus

## Abstract

**Background:**

Left ventricular diastolic dysfunction (LVDD) is a common complication of type 2 diabetes (T2DM), closely associated with obesity and visceral adiposity. The Body Roundness Index (BRI) is a novel anthropometric measure that may better reflect visceral fat distribution, yet its relationship with LVDD in T2DM remains unclear.

**Objective:**

This study aims to investigate the association between BRI and LVDD risk in patients with T2DM, focusing on nonlinear relationships and potential threshold effects.

**Methods:**

This cross-sectional study included 1,317 patients with T2DM. Multivariable logistic regression and generalized additive models (GAM) were used to assess associations, with adjustment for key confounders. Threshold effects were evaluated using a two-step recursive approach, and subgroup analyses were performed.

**Results:**

After full adjustment, each one-unit increase in BRI was associated with a 30% higher risk of LVDD (OR: 1.30, 95% CI: 1.10–1.60, *p* < 0.001). A nonlinear relationship was identified with an inflection point at BRI = 8.1. Below this point, the association was stronger (OR: 1.50, 95% CI: 1.20–1.80, *P* < 0.001). Diabetic kidney disease significantly modified this association (*P* for interaction = 0.02).

**Conclusion:**

BRI is nonlinearly associated with LVDD risk in T2DM, with a threshold effect at BRI = 8.1. The association is stronger in patients with diabetic kidney disease, suggesting that BRI could serve as a valuable marker for the stratification and prevention of LVDD in high-risk populations.

## 1 Introduction

Type 2 diabetes mellitus (T2DM) constitutes a pressing worldwide health challenge. By 2022, the global adult prevalence of diabetes had reached 14%, affecting over 800 million individuals—a more than fourfold increase since 1990 (1). It is projected that the number of affected people will rise to 1.3 billion by 2050 (2), signifying a growing burden on public health and healthcare systems worldwide. Left ventricular diastolic dysfunction (LVDD), a precursor to heart failure associated with poorer clinical outcomes (3, 4), is highly prevalent among individuals with T2DM, with estimates as high as 43% (5). Despite increasing clinical recognition of LVDD, the early detection of this condition remains a significant challenge.

Obesity—particularly the accumulation of visceral adipose tissue (VAT)—is a key driver of cardiometabolic complications in T2DM (6), whose global prevalence continues to rise (7). VAT is not merely a passive energy store but an active endocrine organ. It secretes pro-inflammatory cytokines and adipokines that exacerbate insulin resistance and systemic inflammation, promotes cardiac fibrosis, and contributes to myocardial steatosis (8). These processes, coupled with hemodynamic alterations (such as increased blood volume) induced by obesity, lead to left ventricular remodeling and directly impair myocardial relaxation, establishing a clear pathological link between visceral adiposity and LVDD (9). Accurately quantifying VAT is crucial for risk stratification. The Body Roundness Index (BRI), an innovative anthropometric index developed by Thomas in 2013, provides a more accurate estimate of body fat percentage and visceral adipose tissue volume than traditional indices like BMI (10). A higher BRI, which indicates greater visceral fat, is directly associated with an increased risk of developing LVDD through the pathogenic pathways described above. Currently, it has been confirmed that BRI is associated with type 2 diabetes, type 2 diabetic kidney disease, cardiometabolic syndrome in patients with type 2 diabetes, and even the risk of death (11–16). However, the association between BRI and the risk of LVDD in the T2DM population remains unclear.

This study aims to explore the relationship between BRI and LV diastolic function. Understanding this relationship may facilitate earlier detection and improve the risk stratification of LVDD in patients with T2DM, potentially advancing strategies to enhance clinical outcomes in this population.

## 2 Methods

### 2.1 Study design and participants

This was a single-center, retrospective cross-sectional study. Data were collected from the electronic medical records of the Department of Endocrinology, The Affiliated Hospital of Qingdao University. The study period spanned from January 2010 to July 2024. The study initially included 1,317 hospitalized patients aged 18 and above, diagnosed with T2DM, and who had undergone a complete echocardiogram. Exclusion criteria were carefully defined to mitigate potential confounding factors, including the following:

- Coronary/valvular heart disease or heart failure of class NYHA ≥III
- Reduced ejection fraction (EF < 45%)
- Severe hepatic/renal dysfunction
- Active infections or active autoimmune diseases
- Hematological disorders
- Malignancy
- Lacking echocardiogram results

**Figure 1:**
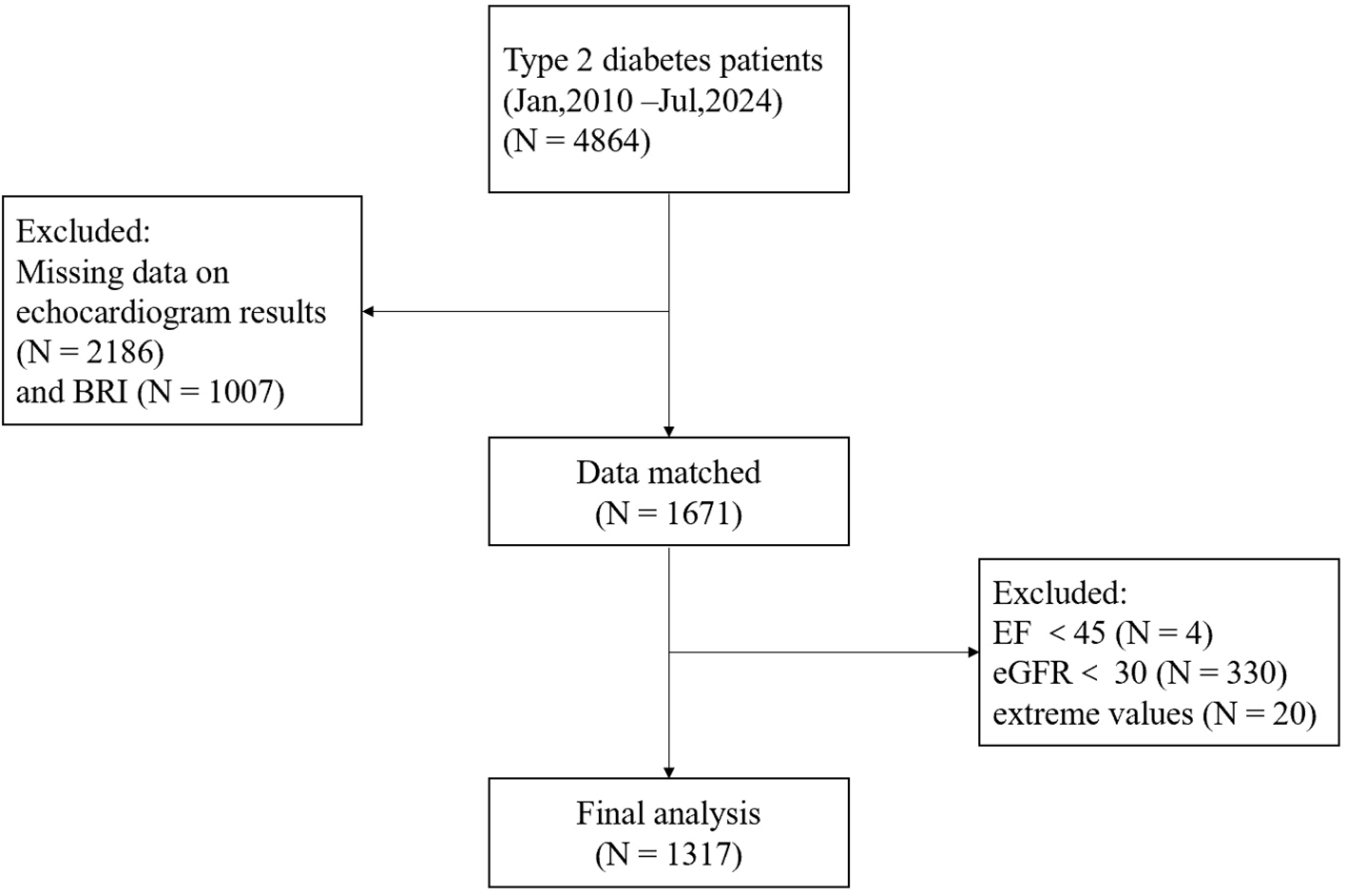
Flowchart of the study population.

The diagnostic criteria for T2DM were defined according to the 2023 Standards of Medical Care in Diabetes of the American Diabetes Association (ADA) (17). Heart failure (HF) was diagnosed in accordance with the 2022 AHA/ACC/HFSA Guidelines (18). Severe hepatic or renal dysfunction was characterized by ALT levels exceeding three times the upper limit or an eGFR lower than 30 mL/min/1.73 m². Additionally, extreme values of BRI were identified and excluded based on a threshold of mean ± 3 standard deviations. After these exclusions, the final analysis consisted of 1,317 participants. **Figure 1** illustrates the application of the inclusion and exclusion criteria.

### 2.2 Ethical considerations

Adhering to the Declaration of Helsinki, informed consent was obtained from all participants. The study received ethical approval from the Ethics Committee of Qingdao University Affiliated Hospital (no. QYFY WZLL 30337).

### 2.3 Calculation of BRI


$$\text{BRI}=364.2-365.5\times \sqrt{1-\frac{{\frac{\text{WC }\left(\text{m}\right)}{2\pi }}^{2}}{0.5\times \text{height }{\left(\text{m}\right)}^{2}}}$$

### 2.4 Diagnosis of LVDD

LV diastolic function was assessed by two experienced echocardiographers using transthoracic echocardiography (Philips EPIQ7) according to the mitral valve flow pattern. Key structural parameters were recorded, namely: left ventricular end-diastolic diameter (LVEDD), left atrial diameter (LAD), interventricular septal thickness (IVST), and left ventricular ejection fraction (LVEF) determined via the biplane Simpson’s approach. Mitral inflow velocities were obtained from apical four-chamber views, with the peak early (E-wave) and late (A-wave) diastolic velocities measured. According to the ASE/EACVI 2016 recommendations, LVDD was defined by either average E/e ratio >14 or E/e’ ratio<14 with E/A ratio< 0.8 (19).

### 2.5 Covariates

#### 2.5.1 Demographic and clinical data

The variables included were gender, age, diabetes duration, and complications.

#### 2.5.2 Anthropometrics

Height, weight, and waist circumference were measured. The calculation of BRI was as described above.

#### 2.5.3 Lifestyle factors

Behavioral risk factors were evaluated through interviewer-administered surveys. Tobacco exposure was defined as the lifetime consumption of ≥100 cigarettes. Regular alcohol use was operationalized as a sustained weekly intake of >30 g for ≥12 consecutive months (20).

#### 2.5.4 Biochemical analyses

Venous blood specimens were collected after overnight fasting and analyzed for serum creatinine, fasting blood glucose, liver function parameters, uric acid, blood lipids, and glycated hemoglobin (HbA1c). All assessments, except for HbA1c, were conducted using a Hitachi 7600 automated analyzer. HbA1c was determined by high-performance liquid chromatography (Bio-Rad Variant II, USA).

#### 2.5.5 Definition of clinical biochemical terms

Hypertension was defined based on any of the following criteria: (1) documented history of antihypertensive medication use, (2) self-reported physician-diagnosed hypertension, (3) mean systolic pressure ≥140 mmHg, and (4) mean diastolic pressure ≥90 mmHg (21). Estimated glomerular filtration rate (eGFR) was calculated using the CKD-EPI formula (22). Diabetic kidney disease was defined per KDIGO 2021 criteria as either (a) ACR ≥30 mg/mg (≥3 mg/mmol) in spot urine sample or (b) eGFR<60 mL/min/1.73 m² sustained >3 months (23). Diabetic peripheral neuropathy (DPN) diagnosis employed a dual-path protocol: (i) clinical evidence required two or more manifestations from sensory symptoms, diminished reflexes, or positive neurological signs consistent with distal symmetric polyneuropathy and (ii) electrodiagnostic confirmation necessitated abnormalities (amplitude/latency/NCV/F-wave) in two or more peripheral nerves (24).

### 2.6 Statistical methods

Continuous variables were reported as mean and standard deviation if they were normally distributed and as median and interquartile ranges (IQRs) if they were skewed. Categorical variables were presented as percentages. We used chi-square (*χ*^2^) test for categorical variables, Student’s *t*-test for normally distributed continuous variables, and Mann–Whitney *U*-test for skewed continuous variables to assess differences in left ventricular diastolic function status among participants. Additionally, differences among BRI quartiles were analyzed using *χ*^2^ test for categorical variables, one-way ANOVA for normally distributed variables, and Kruskal–Wallis H test for skewed distributed variables.

BRI was analyzed both as a continuous variable (per one-unit increment) to assess its detailed association with LVDD risk and as a categorical variable (by quartiles) to facilitate intergroup comparisons.

Multivariable logistic regression analysis was performed to assess independent associations. The model was fitted using the generalized linear model function (glm) in R software, with LVDD as the binary outcome. To ensure the robustness of the model, the assumption of no severe multicollinearity was tested by calculating the variance inflation factor (VIF) for all covariates. A VIF value ≥5 was considered indicative of substantial multicollinearity. Subsequently, we employed logistic regression analysis, systematically adjusting for potential confounders in different models: crude model—no adjustment was made; model I—adjustment was made for basic demographic and clinical factors, including age, sex, and diabetes duration; model II—comprehensive adjustment for an expanded set of variables: age, sex, diabetic duration, uric acid (UA), aspartate aminotransferase (AST), estimated glomerular filtration rate (eGFR), fasting blood glucose (FBG), high-density lipoprotein cholesterol (HDL-C), triglyceride (TG), and hypertension (HP).

To evaluate the nonlinear relationship between BRI and the risk of LVDD, a generalized additive model (GAM) was used. Moreover, subgroup analyses and interaction tests were conducted to investigate the impact of BRI on different patient subgroups. Statistical analyses were performed using R software (version 4.2.0) and EmpowerStats, and two-sided *P*-values<0.05 were considered statistically significant.

## 3 Results

**Table 1** presents the characteristics of the participants in the BRI quartiles: Q1 (2.2–4.7), Q2 (4.7–5.5), Q3 (5.5–6.5), and Q4 (6.5–10.4), and these quartile boundaries were consistently used in our analysis. As presented in **Table 1**, significant differences were observed across BRI quartiles in age, sex, diabetes duration, uric acid, ALT, AST, eGFR, creatinine, fasting blood glucose, high-density lipoprotein cholesterol, and triglycerides. Hypertension prevalence also showed a significant positive association with increasing BRI quartiles, rising from 27.6% (91 cases) in Q1 to 54.9% (174 cases) in Q4 (*P* < 0.001).

**Table 1:** Baseline characteristics of study participants by quartiles of BRI.

BRI quartile	Total (2.2–10.4)	Q1 (2.2–4.7)	Q2 (4.7–5.5)	Q3 (5.5–6.5)	Q4 (6.5–10.4)	*P*-value
*N*	1317	330	336	334	317	
Age (years)	52.7 ± 13.3	54.5 ± 12.1	53.9 ± 12.5	51.7 ± 13.2	50.6 ± 14.8	<0.001
Sex, *n* (%)						<0.001
Male	798 (60.6%)	169 (51.2%)	207 (61.6%)	227 (68.0%)	195 (61.5%)	
Female	519 (39.4%)	161 (48.8%)	129 (38.4%)	107 (32.0%)	122 (38.5%)	
Smoking history, *n* (%)						0.612
Non-smokers	934 (71.0%)	239 (72.6%)	239 (71.1%)	240 (71.9%)	216 (68.1%)	
Smokers	382 (29.0%)	90 (27.4%)	97 (28.9%)	94 (28.1%)	101 (31.9%)	
Alcohol consumption history, *n* (%)						0.652
Non-drinkers	924 (70.4%)	236 (72.2%)	241 (71.7%)	230 (69.1%)	217 (68.5%)	
Drinkers	389 (29.6%)	91 (27.8%)	95 (28.3%)	103 (30.9%)	100 (31.5%)	
Diabetic duration (years), *n* (%)						0.019
<5	563 (45.4%)	112 (36.6%)	149 (46.9%)	152 (47.2%)	150 (51.0%)	
≥5,<10	256 (20.6%)	70 (22.9%)	62 (19.5%)	70 (21.7%)	54 (18.4%)	
≥10	421 (34.0%)	124 (40.5%)	107 (33.6%)	100 (31.1%)	90 (30.6%)	
UA (umol/L)	324.2 ± 95.6	283.8 ± 81.7	317.1 ± 89.6	337.5 ± 92.4	361.1 ± 101.9	<0.001
ALT (U/L, median (IQR))	18.2 (13.7–28.0)	16.0 (12.0–22.0)	17.1 (13.0–25.2)	20.1 (14.3–31.0)	22.0 (16.2–35.9)	<0.001
AST (U/L, median (IQR))	16.2 (13.7–21.0)	15.2 (13.0–19.0)	16.0 (13.0–20.0)	17.2 (14.0–22.0)	18.0 (14.6–25.9)	<0.001
eGFR (mL/min per 1.73 m^2^)	95.7 ± 30.0	99.6 ± 30.7	99.1 ± 28.1	92.8 ± 29.1	91.0 ± 31.2	<0.001
SCr (umol/L)	70.9 ± 20.4	69.3 ± 18.8	71.8 ± 21.0	73.1 ± 19.5	69.1 ± 21.9	0.041
ALB (g/L)	46.0 ± 9.6	46.0 ± 10.3	45.8 ± 9.4	46.1 ± 9.5	46.1 ± 9.1	0.982
HbA1c (%)	9.2 ± 2.2	9.4 ± 2.5	9.1 ± 2.2	9.1 ± 2.0	9.1 ± 2.0	0.244
FBG (mmol/L)	7.8 ± 2.6	7.5 ± 2.6	7.6 ± 2.7	7.8 ± 2.4	8.2 ± 2.7	0.006
LDL-C (mmol/L)	2.9 ± 0.9	2.8 ± 1.0	2.9 ± 0.9	2.9 ± 0.9	2.9 ± 0.9	0.422
HDL-C (mmol/L)	1.2 ± 0.3	1.3 ± 0.3	1.1 ± 0.3	1.1 ± 0.3	1.1 ± 0.3	<0.001
TC (mmol/L)	4.4 ± 1.7	4.2 ± 1.8	4.4 ± 1.7	4.4 ± 1.7	4.6 ± 1.7	0.144
TG (mmol/L, median (IQR))	1.5 (1.0–2.4)	1.1 (0.8–1.6)	1.4 (1.0–2.3)	1.5 (1.1–2.4)	1.8 (1.4–3.1)	<0.001
Hypertension, *n* (%)						<0.001
No	765 (58.1%)	239 (72.4%)	211 (62.8%)	172 (51.5%)	143 (45.1%)	
Yes	552 (41.9%)	91 (27.6%)	125 (37.2%)	162 (48.5%)	174 (54.9%)	
Diabetic kidney disease, *n* (%)						0.819
No	995 (75.6%)	250 (75.8%)	252 (75.0%)	258 (77.2%)	235 (74.1%)	
Yes	322 (24.4%)	80 (24.2%)	84 (25.0%)	76 (22.8%)	82 (25.9%)	
Diabetic retinopathy, *n* (%)						0.317
No	970 (73.7%)	231 (70.0%)	255 (75.9%)	251 (75.1%)	233 (73.5%)	
Yes	347 (26.3%)	99 (30.0%)	81 (24.1%)	83 (24.9%)	84 (26.5%)	
Diabetic peripheral neuropathy, *n* (%)						0.793
No	269 (20.4%)	121 (36.7%)	132 (39.3%)	135 (40.4%)	123 (38.8%)	
Yes	806 (61.2%)	209 (63.3%)	204 (60.7%)	199 (59.6%)	194 (61.2%)	
LVDD						0.854
No	269 (20.4%)	71 (21.5%)	66 (19.6%)	71 (21.3%)	61 (19.2%)	
Yes	1,048 (79.6%)	259 (78.5%)	270 (80.4%)	263 (78.7%)	256 (80.8%)	

Data are given as mean ± standard or median (IQR) or number (percent) as appropriate.

UA, uric acid; ALT, alanine aminotransferase; AST, aspartate aminotransferase; eGFR, estimated glomerular filtration rate; SCr, serum creatinine; ALB, albumin; HbA1c, glycated hemoglobin; FBG, fasting blood glucose; LDL-C, low-density lipoprotein cholesterol; HDL-C, high-density lipoprotein cholesterol; TC, total cholesterol; TG, triglyceride; LVDD, left ventricular diastolic dysfunction.

In our multivariate logistic regression analysis, as shown in **Table 2**, we systematically adjusted for potential confounders to evaluate the association between BRI and the risk of LVDD in several models. When BRI was analyzed as a continuous variable, no significant association was found in the crude model (OR: 1.0, 95% CI: 0.9–1.1, *p* = 0.391). After adjusting for age, sex, and diabetes duration in model I, BRI showed a positive association with LVDD (OR: 1.4, 95% CI: 1.3–1.6, *p* < 0.001). Further adjustment in model II, including variables such as age, sex, diabetes duration, UA, AST, eGFR, FBG, HDL-C, TG, and hypertension, continued to show a positive association (OR: 1.3, 95% CI: 1.1–1.6, *p* < 0.001). These results indicate that after adjustment for potential confounders, higher BRI values were consistently associated with increased odds of LVDD in patients with type 2 diabetes. When analyzed by quartiles, participants in higher BRI quartiles exhibited a graded increase in the prevalence of LVDD in the fully adjusted model (model II), suggesting a dose–response relationship between BRI and LVDD risk (OR vs. Q1: Q2 = 1.1, 95% CI: 0.6–2.1; Q3 = 2.1, 95% CI: 1.1–3.9; Q4 = 2.4, 95% CI: 1.2–4.8).

**Table 2:** Relationship between BRI and LVDD in different models.

Exposure	Crude model OR (95% CI) *P*-value	Model I OR (95% CI) *P*-value	Model II OR (95% CI) *P*-value
BRI (per one unit change)	1.0 (0.9, 1.1) 0.391	1.4 (1.3, 1.6)<0.001	1.3 (1.1, 1.6)<0.001
BRI subgroups			
Q1	1.0	1.0	1.0
Q2	1.1 (0.8, 1.6) 0.550	1.4 (0.8, 2.2) 0.236	1.1 (0.6, 2.1) 0.665
Q3	1.0 (0.7, 1.5) 0.935	1.8 (1.1, 3.0) 0.020	2.1 (1.1, 3.9) 0.027
Q4	1.2 (0.8, 1.7) 0.474	3.3 (1.9, 5.6)<0.001	2.4 (1.2, 4.8) 0.017

Models were adjusted for covariates as follows: crude model—unadjusted; model I—adjusted for age, sex, and diabetic duration; model II—adjusted for variables in model I plus UA, AST, eGFR, FBG, HDL-C, TG, and hypertension.

**Figure 2:**
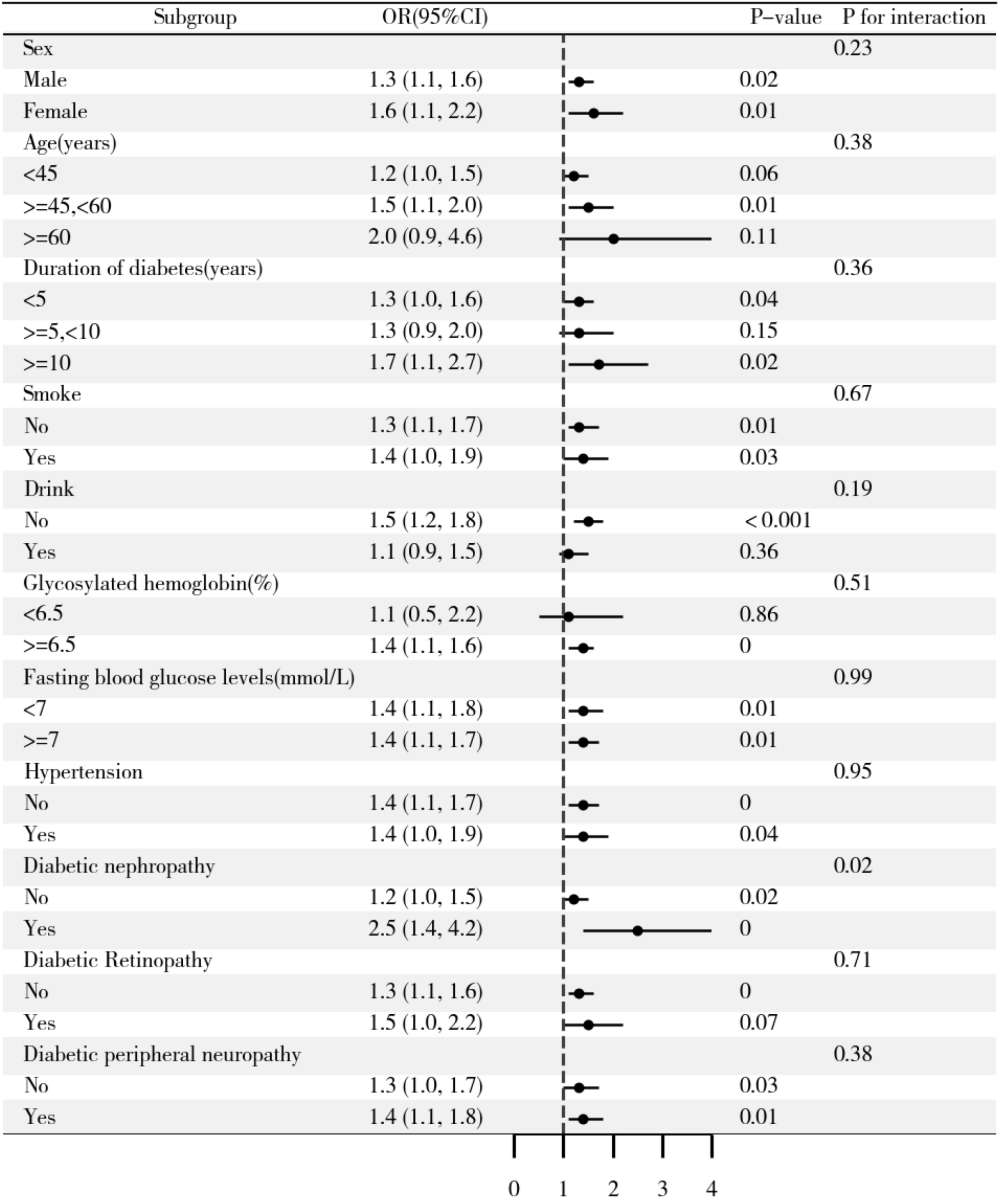
Results of the subgroup analyses. The analysis was adjusted for age, sex, diabetic duration, UA, AST, eGFR, FBG, HDL-C, TG, and hypertension, excluding the variable specific to each subgroup under investigation.

To assess the consistency of the association between BRI and LVDD across different patient subgroups, interaction tests and stratified analyses were conducted using a fully adjusted model, as shown in **Figure 2**. The results indicated that the positive association between elevated BRI and LVDD was not uniform across all subgroups. Specifically, no statistically significant association was observed (*p* > 0.05) in certain cohorts, including individuals younger than 45 or older than 60 years, those with a diabetes duration of 5–10 years, alcohol consumers, participants with glycated hemoglobin<6.5%, and those diagnosed with diabetic retinopathy.

Notably, no significant interactions were found in the subgroups of sex, age, diabetes duration, smoking, alcohol consumption, glycated hemoglobin, fasting blood glucose, hypertension, diabetic retinopathy, and diabetic peripheral neuropathy, suggesting that these variables did not affect the positive correlation between BRI and the risk of LVDD (all interaction *p*-values >0.05). However, a significant interaction was observed based on DKD status (*P* for interaction = 0.02). Among patients with DKD, each one-unit increase in BRI was associated with a 1.5-fold increase in the risk of LVDD. Conversely, among those without DKD, a comparable increase in BRI was associated with 20% increase in the risk of LVDD. These findings suggest that DKD may significantly modify the association between BRI and LVDD, highlighting its potential role in risk stratification and personalized management strategies for diabetic patients.

**Figure 3:**
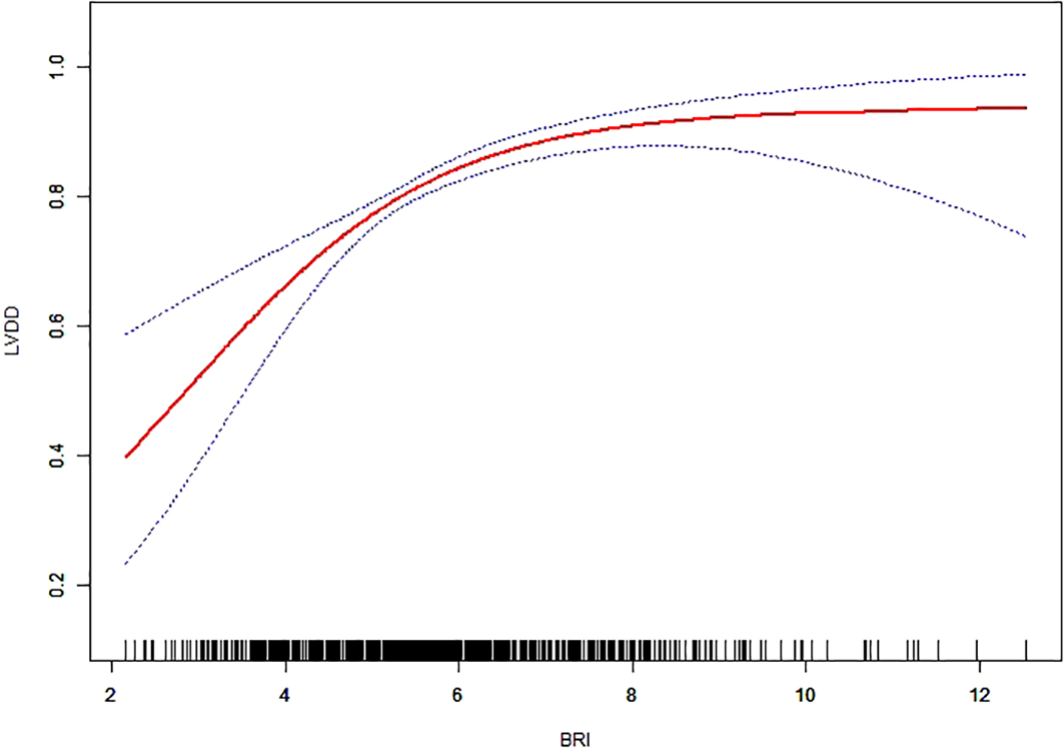
Dose–response association between BRI and LVDD. This generalized additive model (GAM) plot illustrates the relationship between BRI (X-axis) and the risk of LVDD (Y-axis). The area between the upper and lower dashed lines is represented as 95% CI. Each point shows the magnitude of the BRI and is connected to form a continuous line. Age, sex, diabetic duration, UA, AST, eGFR, FBG, HDL-C, TG, and hypertension were adjusted.

**Figure 4:**
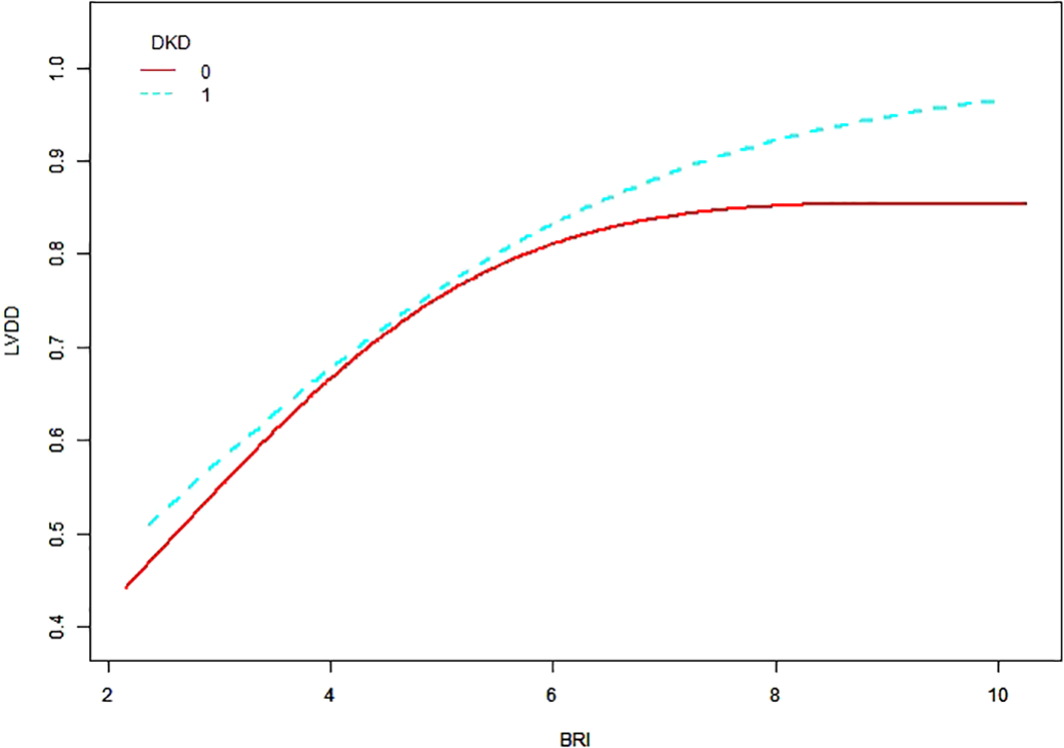
Association between BRI and LVDD stratified by DKD. This generalized additive model (GAM) plot illustrates the relationship between BRI (X-axis) and the risk of LVDD (Y-axis), stratified by DKD status. The solid red line represents the fitted relationship for patients without DKD (DKD = 0), while the dashed blue line represents the relationship for patients with DKD (DKD = 1). The smooth term for BRI was significant (all *P* < 0.05; edf > 1) in both DKD strata. Age, sex, diabetic duration, UA, AST, eGFR, FBG, HDL-C, TG, and hypertension were adjusted.

To investigate the potential nonlinear relationship between BRI and the risk of LVDD, we utilized a generalized additive model (GAM) with smooth curve fitting. After adjustment for age, sex, diabetes duration, UA, AST, eGFR, FBG, HDL-C, TG, and hypertension, a significant positive association was observed between BRI and LVDD risk (**Figures 3**, **4**).

To further quantify this relationship, a threshold analysis was performed using a segmented regression model and a recursive algorithm (**Table 3**). An inflection point was identified at BRI = 8.1 (likelihood ratio test *p* = 0.026). Below this threshold, each one-unit increase in BRI was associated with a 50% elevated risk of LVDD (OR = 1.5, 95% CI: 1.2–1.8; *p* < 0.001). Beyond this point, however, the association was no longer statistically significant (OR = 0.5, 95% CI: 0.2–1.2; *p* = 0.114).

**Table 3:** Threshold effect of BRI on LVDD and its stratification based on DKD.

Model	OR (95% CI)	*P*-value
Model I (linear effect)		
One-line effect	1.3 (1.1, 1.6)	<0.001
Model II (nonlinear effect)		
Threshold	8.1	
Effect for<8.1	1.5 (1.2, 1.8)	<0.001
Effect for >8.1	0.5 (0.2, 1.2)	0.114
Log-likelihood ratio		0.026
Stratified by DKD		
Yes		
Threshold	3.5	
Effect for<3.5	23.0 (0.5, 991.2)	0.102
Effect for >3.5	2.2 (1.2, 3.9)	0.006
Log-likelihood ratio		0.220
No		
Threshold	8.1	
Effect for<8.1	1.4 (1.1, 1.7)	0.002
Effect for >8.1	0.4 (0.2, 1.1)	0.076
Log-likelihood ratio		0.025

Exposure—BRI (per one unit increase), outcome—LVDD. Adjusted for age, sex, diabetic duration, UA, AST, eGFR, FBG, HDL-C, TG, and hypertension. *P* < 0.05 for log-likelihood ratio test indicates that the nonlinear model (model II) is significantly different from the linear model (model I).

OR, odds ratio; CI, confidence interval.

Subgroup analysis stratified by DKD status indicated that patients with DKD exhibited a consistently higher risk of LVDD at comparable BRI levels. In this subgroup, the inflection point occurred at BRI = 3.5, although it did not reach statistical significance (log-likelihood ratio test *p* = 0.22).

## 4 Discussion

Diabetic cardiomyopathy, with left ventricular diastolic dysfunction (LVDD) as one of its earliest functional manifestation, represents a critical pathway to heart failure and increased mortality in type 2 diabetes (T2DM) (25–29). Early identification of individuals at high risk for LVDD is therefore a pressing clinical priority. Obesity, particularly visceral adiposity, is a key driver of cardiac dysfunction in T2DM, acting through hemodynamic overload, chronic inflammation, and metabolic dysregulation (30, 31). The Body Roundness Index (BRI) has emerged as a superior anthropometric indicator of visceral adipose tissue volume compared to conventional measures like BMI (32, 33). However, its specific relationship with LVDD in the T2DM population remained uncharacterized.

In this cross-sectional study, we utilized GAM to examine the relationship between BRI and LVDD in patients with T2DM. The analysis revealed a significant positive nonlinear association. The risk of LVDD increased sharply with rising BRI until it reached an inflection point around 8.1. Beyond this point, the risk curve flattened, indicating that further increases in BRI were associated with a much smaller rise in risk.

This identified inflection point may hold a direct clinical relevance. It proposes a potential threshold for risk stratification. For individuals with a BRI below this point, there is a critical opportunity for primary prevention. Lifestyle and metabolic interventions at this stage may effectively slow the rapid rise in LVDD risk. Conversely, exceeding this threshold might identify patients who have transitioned into a higher-risk state where the risk curve flattens. For these individuals, treatment should focus more aggressively on controlling heart and metabolic health to lower their already high risk. However, the limited number of high-BRI participants in our study means that the exact inflection point requires confirmation in larger, future studies with representation across all BRI values.

The observed nonlinear association between BRI and LVDD can be mechanistically explained through multiple interlinked pathways driven by visceral adiposity. First, excess visceral fat can release high levels of pro-inflammatory molecules (such as TNF-α and IL-6) and reduce anti-inflammatory adiponectin (34, 35). This chronic low-grade inflammatory state promotes myocardial fibrosis and stiffness, directly impairing the heart’s ability to relax during diastole. Second, visceral fat is a major site for ectopic fat storage and increased insulin resistance. The consequent lipotoxicity and changes in cardiac energy metabolism contribute to fat accumulation and dysfunction in heart muscle cells. Concurrently, hyperinsulinemia can boost sympathetic nervous system activity and cause the kidneys to retain more sodium, which increases the heart’s workload (36, 37). Third, obesity leads to significant hemodynamic alterations. The rise in blood volume and cardiac output induces structural remodeling of the left ventricle and elevates its filling pressure, both of which are key factors in the development of LVDD (38, 39). The fact that LVDD risk no longer continues to rise steeply beyond a certain BRI point may imply that the underlying disease mechanisms may have a limited capacity to cause further harm. Beyond a certain point of fat accumulation, further activation of inflammatory, metabolic, and neurohormonal pathways may cause progressively less additional harm to diastolic function. Two explanations may account for this: on one hand, the involved pathways might be working at their peak capacity; on the other hand, the body might have started to adapt in response.

Furthermore, our study revealed that DKD significantly modified the BRI–LVDD association. Patients with DKD exhibited a higher risk of LVDD across all BRI levels. This effect occurs because the disease processes in the kidney and heart are deeply connected. Specifically, DKD leads to impaired sodium excretion and overactivates the renin–angiotensin–aldosterone system (RAAS) (40). This, in turn, worsens fluid retention and high blood pressure, placing a greater burden on the heart. Additionally, the accumulation of uremic toxins and a state of microinflammation further promote endothelial dysfunction, vascular stiffness, and myocardial fibrosis, collectively amplifying the diastolic impairment initiated by visceral obesity (41–43).

This study possesses several notable strengths. To our knowledge, it is the first to evaluate the association between BRI and LVDD specifically in a population with type 2 diabetes, providing novel insights into the relationship between central obesity and cardiac function using this relatively new anthropometric measure. Moreover, the application of smooth curve fitting enabled the identification of a nonlinear relationship and a precise inflection point between BRI and LVDD.

Several limitations of this study should also be acknowledged. Firstly, the cross-sectional design cannot establish causality between BRI and LVDD. Secondly, the exclusion of a large number of cases due to missing data and the exclusion of extreme BRI values may respectively affect the generalizability of our research findings and the precise estimation of the association in the high BRI range. Thirdly, although multiple factors have been adjusted, the possibility of residual confounding still cannot be completely ruled out. Finally, the single-center recruitment and specific inclusion criteria may limit the external validity of our results to the broader type 2 diabetes population.

## 5 Conclusion

In conclusion, this study demonstrates a significant positive association between BRI and the risk of LVDD among patients with type 2 diabetes. Using smooth curve fitting, we identified a nonlinear relationship, with threshold analysis further revealing an inflection point in this association. Furthermore, DKD was found to be a key effect modifier, with patients exhibiting DKD showing substantially elevated LVDD risk at comparable BRI levels. These findings highlight the potential clinical utility of BRI to improve risk stratification and guide preventive strategies against LVDD in type 2 diabetes, especially in high-risk subgroups such as those with DKD. Further longitudinal studies are needed to confirm causality and elucidate the underlying mechanisms, particularly the mediating role of cardiorenal pathways.

## Data Availability

The raw data supporting the conclusions of this article will be made available by the authors, without undue reservation.
